# Seizure first aid in the community: current situation, suggestions, and the role of the general practitioner in seizure management

**DOI:** 10.1186/s42494-025-00202-w

**Published:** 2025-03-03

**Authors:** Mengtian Sun, Fanlong Meng, Zheng-yan-ran Xu, Yi Guo

**Affiliations:** 1 Zhejiang Chinese Medical University, Hangzhou, 310053 China; 2 Yunhe Street Community Health Service Center, Linping District, Hangzhou, 311102 China; 3 Department of Neurology, Epilepsy Center, The Second Affiliated Hospital Zhejiang University School of Medicine, Hangzhou, 310009 China; 4 Department of General Practice, The Second Affiliated Hospital Zhejiang University School of Medicine, Hangzhou, 310009 China

**Keywords:** Seizure, First aid, Community, Training, General practitioner

## Abstract

The unpredictability of seizures underscores the importance of timely recognition and intervention for optimal prognosis. Seizure first aid (SFA) is an essential skill for community members. We reviewed the literature to assess the challenges and explore potential solutions for effective SFA implementation in community settings. The findings reveal that the knowledge of SFA varies significantly among different groups and countries. There are common misunderstandings, such as point therapy, unnecessary ambulance calls, putting objects into the mouth, inappropriate administration of anti-seizure medications, and performing cardiopulmonary resuscitation. Effective SFA training content includes ensuring the safety of patients, avoiding restraint, using lateral position, clearing the respiratory tract, avoiding placing objects into the mouth, recording details, and seeking for professional help. Training methods range from hospital-based courses to community center workshops and online platforms. General practitioners play a pivotal role in epilepsy management and should be actively involved in SFA training initiatives. Therefore, the development of targeted, diverse, and comprehensive training and evaluation strategies, along with collaborative efforts from the whole society, is essential to improve the level and effectiveness of community SFA.

## Background

Epilepsy, a chronic neurological disorder characterized by recurrent seizures, affects over 70 million individuals globally [1]. A significant proportion of the population will experience at least one seizure in their lifetime (approximately 10%) [2–4], with an pooled point prevalence of active epilepsy at 6.38 per 1000 individuals [5]. Emotional stress, sleep deprivation, exposure to flashing lights, and the use of drugs or alcohol can trigger seizures in people with epilepsy (PWE). Seizures present with various symptoms, including convulsions, loss of consciousness, abnormal movements, and sensory disturbances [6]. The potential consequences of seizures can be serious, including fractures, dislocations, soft tissue injuries, head trauma, aspiration pneumonia, and the rare but severe risk of sudden unexpected death [7, 8].

Given the unpredictable nature of seizures and their potential occurrence in any setting, from homes to public spaces, prompt and appropriate responses are crucial from family caregivers, general practitioners (GPs), and the general public, especially in cases of cluster seizures (CSs) or status epilepticus (SE). Timely recognition and intervention are essential for improving the prognosis of PWE, helping to prevent exacerbations or complications [9], and allowing for the timely provision of professional medical assistance. This article reviews the current state and challenges of seizure first aid (SFA) in community settings through a literature review and discusses the role of general practice medicine in it.

## Current situation

### First aid situations in different countries

#### Medical personnels’ knowledge and practices

The knowledge of seizure first aid and correct answer rates among different medical personnel are summarized in Table 1 [10–16].

**Table 1:** Knowledge of seizures first aid in different literature among different types of medical personnel (%)

First aid knowledge of seizures section items	Chomba et al. [10]	Dayapoğlu et al. [11]	Martino et al. [12]	Assadeck et al. [13]	Sun et al. [14]	Yu et al. [15]	Zhao et al. [16]
Area	Lusaka and Monze, Zambia	Erzurum, Turkey	Apulian, Italy	Niamey, Niger	Zhejiang, China	Zhejiang, China	Henan, China
Year	2006	2014	2016	2019	2022	2021	2016
Target population (*n*)	Healthcare workers (276)	Clinical nurses (96)	Healthcare workers (154)	Nurses (98)	GPs (123)	Nurses (235)	Medical staff (219)
	Urban and rural districts	Tertiary hospital	Tertiary hospital		Community health service center	Tertiary hospitals	Tertiary hospitals
Remove sharps around to prevent injuries			89.4	3.1	90.2	96.2	94.5
Clear airway			22.9	6.1	73.2	94.0	88.1
Spill cold water on his/her face to promote wakefulness		15.3		3.1	17.9	94.0	84.0
Press his/her mouth, feeding anti-seizure medications			11.3		50.4	71.5	63.0
Put something in his mouth	58.8		54.0				
Turn him to the side	92.6		90.6	6.1			
Loosen anything on his neck			52.4				
Hold him/her down	38.6		11.6		37.4	91.5	84.9
Give oxygen	29.4						
Smell an onion or cologne		20.0					
Press philtrum and Hukou					44.7	85.5	64.8
Artificial respiration and external cardiac compression					26.8	54.9	52.1
Read the Quran				1.0			

In Zambia [10], 58.8% of healthcare workers reported a willingness to place a hard object into the patient's mouth, while over 35% indicated they would restrain the patient. Additionally, 29.4% of respondents stated they would administer oxygen. Surprisingly, as of 2014, 15.3% of clinical nurses in Turkey were found to splash cold water on the patient's face, and one out of every five nurses would use olfactory stimuli (onion or cologne) to induce wakefulness in PWE [11]. Martino et al. [12] found that 22.9% of respondents believed cardiopulmonary resuscitation (CPR) should be initiated during a seizure, 54.0% of respondents advocated for placing something hard in the patient's mouth, and 11.3% thought medicine should be administered orally during a seizure. Other endorsed first-aid measures included turning the patient on his/her side (90.6%), clearing the airway (71.0%), loosening anything around the neck (52.4%), and holding the person down (11.6%). In Niger [13], 35.7% of nurses expressed uncertainty about how to respond during an epileptic seizure. Only 6.1% of respondents correctly identified the appropriate actions (lying the patient on their side, clearing the airway if necessary, and diazepam injection). In 2022, Asadi-Pooya et al. [17] conducted a study at Namazi Hospital in Iran to assess healthcare workers' perspectives and knowledge regarding SFA for PWE. More caregivers than nurses and physicians provided incorrect answers to most questions (for example, not timing the seizure, not loosening the clothing around the neck, and not rolling the patient onto their side if unconscious).

In contrast, the majority of medical staff in China have a fundamental understanding of SFA and are equipped to respond during seizures [14–16]. However, only a few of GPs have mastered accurate SFA skills. Compared to staff in tertiary hospitals, GPs demonstrated a lower level of SFA knowledge, and most of them fail to master SFA adequately. Their scores for each item of first-aid knowledge were lower than those of medical staff from the general hospitals. For instance, a substantial portion of GPs [14] reported using inappropriate interventions, such as forcefully hold down the limbs (nearly two-thirds), and selected first-aid methods like artificial respiration and CPR (73.2%), and splashing cold water on the patient’s face to promote wakefulness (82.1%) [14]. Furthermore, half of GPs (50.4%) usually pressed on the patient's mouth, attempting to administer anti-seizure medications (ASM) orally during a seizure [14].

#### Public knowledge and practices

A summary of SFA knowledge among the general public in different studies is shown in Table 2 [18–24].

**Table 2:** Knowledge of seizures first aid in different among different the public personnel (%)

First aid knowledge of seizures section items	Kankirawatana [18]	Pandian et al. [19]	Bozkaya er al [20]		Goel et al. [21]	Ekeh et al. [22]		Zhao et al. [23]				Cofano et al. [24]
Country	Thailand	India	Turkey		India	Nigeria		China				Grenada
Year	1999	2003–2004	2005		2010–2011	2015		2022				2015
Target population (*n*)	Teachers (284)	High school students (1213)	Children (851)		Students (177)	Medical Students		University students				Adult residents (200)
			Pretest	Posttest		Basic (121)	Clinical (111)	Education (780)	Medicine (802)	Science and engineering (794)	Total (2376)	
Remove dangerous objects to prevent injuries			25.5	46.3	61.0	57.9	90.1*	71.7^a^	89.4^b^	83.0^c^	81.4	95.5
Protecting the head			30.1	54.2								87.0
Keeping the airway open			31.3	45.1				50.4^a^	68.3^b^	60.1^c^	59.7	
Pulling the tongue back			17.3	23.3								
Press his/her mouth, feeding ASMs								65.6^c^	68.2	73.0^a^	69	
Putting something between the teeth	73.0		19.1	36.4	40.7	36.4	25.2	59.4	45.1^b^	61.5	55.4	17.7
Laying the patient			21.3	30.8								
Putting off the clothing			11.1	10.5	32.2	55.4	76.6*					90.0
Smelling onion or cologne			26.5	15.3								
Calling help			43.7	50.9	74.0							95.5
Waiting for the seizure to end			25.2	18.8								
Run away						7.5	0.9*					
Turn him/her to the side					17.5	24.0	44.1*					77.5
Help him/her stand after the seizure						50.4	34.4*					
Reassure him/her after the seizure						43.9	57.7					
Sprinkle water over the face		23.9						92.4^c^	94.1	96.5^a^	94.4	
Hold him/her down	30.2							63.7^c^	67.7^a^	72.8^a^	68.1	41.5
Note the time at which the seizure starts												83.5
Press philtrum and Hukou								55.6^c^	60.3	61.7^c^	59.3	
CPR								85.4	88.3	89.3	87.7	

a/b/c: represents the figures in education majored students(a)/medical students(b)/science and engineering majored students(c) were significantly different from students in the other two groups (*P* < 0.05)

^*^ Correlation was significant at the 0.05 level (2 tailed)

In Thailand [18], nearly half of the teacher respondents (45%) had performed first-aid seizure management. Most respondents (86.4%) were unaware of the proper measures for first-aid management of seizure. Many teachers employed potentially harmful interventions for first-aid seizure management, such as inserting objects (a spoon, a gag or cloth) into the mouth of a person having a seizure (73%), attempting to hold the person down (30.2%), and placing patients on their backs (18.9%). Only 11.4% of the respondents gave the proper first-aid management.

In India [19], 23.9% of respondents would sprinkle water on the face of PWE.

In Ankara, Turkey, a significant increase in SFA knowledge was observed among schoolchildren following an epilepsy education program [20]. However, a considerable proportion (19.1% to 36.4%) still believed it was necessary to "put something between the teeth".

In Nigeria [22], 57.9% of basic students and 90.1% of clinical students indicated they would protect a person having a seizure from injury. Additionally, 36.4% of basic students compared to 25.2% of clinical students indicated they would put something in the person's mouth. Meanwhile, 55.4% of basic students and 76.6% of clinical students would correctly loosen any constricting items around the person's neck. Only 7.5% of basic students and 0.9% of clinical students would run away. Similarly, 24.0% of basic students and 44.1% of clinical students would turn the person to the lateral position. 50.4% of basic students compared to 34.3% of clinical students would help the person stand after the seizure. Additionally, 57.7% of clinical students knew to reassure the person after the seizure, compared with 43.9% of basic students.

In China, Zhao et al. [23] found that no significant difference in knowledge levels regarding SFA between students specializing in clinical medicine and those in other fields (education, science and engineering majors). In other words, compared to preclinical students, clinical students did not possess more first aid expertise.

In Grenada [24], the majority of adult volunteers (87.0%) correctly acknowledged that a victim's head should be cushioned, and 83.5% knew the time when the seizure starts should be noted. However, 41.5% of respondents erroneously believed that a victim should be held down, and 17.7% indicated that medicine should be put into a victim's mouth during a seizure.

#### Cultural differences influence on SFA practices

Cultural beliefs and practices significantly influence SFA approaches globally. Due to cultural differences, pressing the philtrum or the region between the thumb and index finger (Hukou) is believed to help comatose individuals wake up in Chinese tradition. More than half of GPs believe that pressing these traditional Chinese medicine acupressure points is the correct treatment, a belief similar to that of basic-level medical personnel in the Hunan study [25]. In Niger [13], religious practices, such as reciting the Quran for patients, are integrated into patient care, reflecting local faith-based traditions. In Turkey [11, 20], schoolchildren and even nurses may olfactory stimuli, such as onions or cologne to stop the seizure. The use of cologne is linked to the history of the Napoleonic period. In India, where public education is neglected, various improper and even dangerous seizure-control techniques are frequently adopted, such as burning patients, putting irritants in their eyes, placing patients over fires, giving them cow urine, and making them hold a bunch of keys [19, 26, 27]. Cattle urine is called "holy water" in India, and drinking cow urine is considerd to cure many diseases. These facts highlight the influence of deeply entrenched cultural beliefs on health practices.

### Misconceptions

Moeller et al. found that fewer than 15% public could correctly identify focal seizure symptoms and many people wrongly thought all seizures are like tonic-clonic ones, which are misrepresented in media with incorrect first aid [28]. The public lacks awareness of SFA and uses improper measures [18]. Targeted educational initiatives are crucial to address these misconceptions (such as point therapy, unnecessary ambulance calls, putting something in the mouth, orally administration of ASMs, and performing CPR, etc.) and promote the adoption of evidence-based SFA practices.

#### Point therapy

In traditional Chinese medicine, point therapy is a unique treatment method that regulates the body's qi, blood, and organ functions by stimulating specific acupuncture points through techniques such as acupuncture, cupping, and massage. It is noteworthy that pressing the philtrum and Hukou during a seizure is a common misconception belief among Chinese. This belief may stem from the popularity of traditional Chinese medicine, making point therapy more common in Chinese medical practices. Educational levels and workplace environments may also contribute to the prevalence of this misconception.

#### Unnecessary ambulance calls

A survey conducted in Jazan, Saudi Arabia, revealed that the most prevalent misunderstanding was the belief that an ambulance should always be called immediately, regardless of the seizure's specifics (88.3%) [29].

#### Putting something in the mouth

Notably, another prevalent misconception is placing objects in the patients' mouths to prevent seizure-related harm [30]. Studies have shown that 60–90% of the public respondents from Saudi Arabi [29, 31], Iran [32], Turkey [33], Kuwait [34], and China [35, 36] would put something in the mouth to prevent tongue biting or swallowing, often due to television shows and other forms of mass media [36]. Unfortunately, the media continues to propagate thses misconceptions. Only 34.3% of students in a recent study on epilepsy knowledge among university students in the Jazan region responded similarly [37]. The discrepancy is likely explained by the education level of individuals. This action is unnecessary: during a seizure, individuals cannot swallow their tongue, and attempting to put something in their mouth could risk jaw or teeth injure [2]. A study conducted in the United States (US) from 2000 showed 41% of PWE thought it was acceptable to put a solid object in their mouth during a seizure; in a 2020 poll in Germany, that number dropped to 5% [38]. According to Lang et al. [38], this shift is likely to reflect the impact of patient education over two decades.

#### Feeding anti-seizure medications

In numerous studies, a minuscule proportion of individuals were aware that ASMs should not be administered orally during a seizure due to the risk of swallowing difficulties and choking hazards [2]. Specifically, 26.8% of individuals in Jazan [29], 41.4% of Saudis [31], 45.9% of Iranians [32], and 81.5% of Grenadians [24] were aware of this measure.

#### Cardiopulmonary resuscitation

CPR is unnecessary during a seizure, as individuals generally regain spontaneous respiration after the seizure ends. Similar conclusions were reported in two studies conducted in Saudi Arabia [29, 31], where some participants remained unaware of this fact. This is alarming because it suggests that many individuals might perform CPR on people experiencing seizures who are still breathing.

In terms of first aid knowledge perception, significant disparities exist among different groups. These differences can be attributed to multiple factors, including educational background, gender, age, community background, and cultural elements [39, 40]. Fear of experiencing a seizure and uncertainty about how to respond are common barriers to effective SFA [41]. Our findings indicate that the public may overtreat patients with self-limiting seizures due to the fear of SE and its potential consequences. Nonetheless, some misconceptions persist even among medical professionals.

Despite the general acknowledgment of the importance of SFA knowledge, challenges remain within the community. According to the study, approximately 80% to 85% of residents lacked adequate knowledge, with only 32.3% of participants demonstrating a good level of first-aid knowledge [39]. This suggests that the dissemination of first-aid knowledge still requires further enhancement. Additionally, many communities lack professional resources for first aid training [21], particularly in more remote areas. Limited training opportunities for community medical staffs have hampered the delivery of first aid knowledge. First aid education for teenagers is relatively scarce, and many schools do not adequately cover first aid knowledge in their curriculum. Clinical students in college did not possess more knowledge about first aid than preclinical students [23]. Currently, the majority of material in Chinese textbooks on neurology, emergency medicine, and disaster medicine focuses on patient rescue and treatment strategies for SE patients but omit information on pre-hospital first aid skills for isolated seizures. This underscores the need to enhance future pre-hospital first aid instruction for medical students regarding isolated seizure episodes. Thus, there is a critical need to bolster first aid education for adolescents to cultivate their emergency response capabilities.

### Training content

When comparing the SFA guidelines from the Association Against Epilepsy of the China [42], the US [2], Australia [43], and Canada [44]. We found that the initial response is crucial. Witnesses to a seizure should remain calm and prioritize the following steps:

#### Protecting the patient

First and foremost, ensure the patient's safety by gently laying them flat on the ground, clearing the surrounding area of any dangerous objects, such as sharp or hard items, to prevent injury. Simultaneously, loosen their collar and belt, and assist in removing glasses, dentures, or anything that could obstruct their airway.

#### Avoiding restraint and forced movement

Do not attempt to restrain the person or move the patient during the seizure, as this can result in bone fractures or other injuries. Allow the patient to move naturally until the episode concludes.

#### Side lateral position

Gently turn the patient's head to one side to maintain a clear airway. The risk of aspiration increases during a convulsion due to suppressed protective reflexes for the airway. Changing the position can reduce the risk of aspiration and may also alleviate breathing difficulties by allowing fluids to drain more easily [45].

#### Cleaning the respiratory tract

If secretions or vomit are present in the mouth, carefully clean them up, avoiding reaching directly into the mouth to minimize the risk of being bitten.

#### Avoiding placing objects in the mouth

Some may mistakenly believe that placing objects in a patient's mouth can prevent tongue biting. However, this is practically unnecessary and could even cause suffocation.

#### Recording time and symptoms

If possible, family members and witnesses should record the details of seizure, including symptoms, start time of the seizure onset, and duration. This information is valuable for medical staff in subsequent evaluations.

#### Emergency

Call emergency services if the seizure lasts longer than 5 min for a convulsive seizure, if it occurs repeatedly without full recovery between episodes, if it occurs in water, if the person’s breath is impaired, or if it is the person's first seizure, or if the individual is injured or pregnant [46, 47]. Community members should be familiar with these symptoms to act promptly.

#### Training method

The shift in epilepsy awareness is partly due to public education efforts by the Anti-epilepsy Association and experts [48] who have organized talks, webcasts, books, and events on International Epilepsy Caring Day and other occasions over the past few years. SFA training is available through hospitals, clinics, community centers, and online platforms. Hospitals provide professional courses with theory and practice, while community centers offer basic skills training. Online resources, including courses and simulations, offer flexible and accessible learning for all.

For SFA training, many countries have implemented various projects. Most scholars discovered that targeted training enhances both basic knowledge and the response to epilepsy seizure within communities. As shown in Table 3, a study by Bozkaya et al. [20] compared the responses of students in rural and urban areas to raise awareness and attitudes toward epilepsy, showing an improvement through educational programs. One study found that after participating in a brief epilepsy education session, children's understanding of epilepsy improved [49]. In a randomized controlled trial, Martiniuk et al. [50] found that an epilepsy education program increases knowledge and fostered positive attitudes toward epilepsy among fifth-grade students. Goel et al. [21] conducted a comprehensive epilepsy education program for school teachers in Chandigarh, India, which included audio-visual material covering basic aspects of epilepsy. A comparison of knowledge and skills regarding first-aid management of epilepsy, based on pre- and post-training questionnaire scores, showed significant improvements in various domains. The Saudi Epilepsy Society organized a citywide awareness campaign in Riyadh, Saudi Arabia, in 2013, administering questionnaires to the same individuals before and after campaign. The epilepsy awareness campaign significantly increased general knowledge about epilepsy and improved attitudes towards PWE [51]. In addition, similar positive results have been observed in previous interventional research with trainee teachers in Nigeria [52] and Italian primary school teachers [53]. An educational program for students in grades 8–10 resulted in a dramatic increase in understanding of epilepsy, from 11% to 93% [54]. Similarly, in Lebanon, the percentage of participants attempting to stop tongue biting during a seizure dropped from 64.4% to 13.7% after an educational intervention on epilepsy [55]. In Malaysia, the addition of the SMS-based Epilepsy Education System to the conventional epilepsy education module has been effective in improving the health-related quality of life status of PWE [56]. Educational strategies, including educational brochures targeted at college-aged students, seminars for the general population assessed via pre- and post-education questionnaires, door-to-door interviews, and television campaigns, have all demonstrated effectiveness in community-based education [57].

**Table 3:** Seizure first aid training method

Authors	Year	Country	Sample (*n*)	Variables	The findings of the study
Bozkaya et al. [20]	2005	Turkey	Students (851)	Educational sessions comprising simulated patient practice and case-based discussions. Teaching materials included seizure videos	A significant increase in knowledge and positive attitudes towards epilepsy was observed. Students at higher socioeconomic levels demonstrated better performance on both pre- and post-tests
Caveness et al. [49]	1949–1979	The United States	Adult (1539)	At 5-year intervals, the American Institute of Public Opinion obtained responses to epilepsy-related questions from representative adult population members	The proportion of adverse responses was reduced. In each of the seven surveys, the most favorable opinions were among the better educated, better employed, younger, and urban population members
Martiniuk et al. [50]	2006	Canada	Students in grade 5 (783)	An epilepsy education program	Increased awareness and positive attitude towards epilepsy among students
Goel et al. [21]	2012	India	Teachers (85)	A comprehensive epilepsy education program including audio-visual material on basic aspects of epilepsy	Significant improvements in the knowledge and skills regarding first-aid management of epilepsy (P < 0.05)
Alaqeel et al. [51]	2013	Saudi Arabia	The public (2118)	A citywide awareness campaign	Significantly improved general knowledge of epilepsy and attitude towards PWE
Eze et al. [52]	2013	Nigerian	Trainee teachers (226)	Health education	Enhanced knowledge, awareness, and attitude about epilepsy to strengthen emergency care for epilepsy
Mecarelli et al. [53]	2013	Italian	Primary school teachers (582)	An educational campaign	A special educational program was effective in improving basic knowledge of epilepsy and managing seizures
Kolar Sridara Murthy et al. [54]	2014–2015	India	Children from 8th-10th grades (70)	An educational program and information, education, and communication (IEC) materials	93% of the students had sufficient understanding, up from 11% prior to the program. Health education programs for schoolchildren are crucial for bringing changes in their attitude, behavior, and practices
Abou Khaled et al. [55]	2018	Lebanon	Teachers and counselors (73)	An educational epilepsy intervention with Powerpoint presentations and SFA of audiovisual materials, flyers and videos	The percentage of participants attempting to stop tongue biting dropped from 64.4% to 13.7%
Lua et al. [56]	2013	Malaysia	Patients (130)	Printed versus SMS-based education intervention	The addition of the Mobile Epilepsy Education System to the conventional epilepsy education module has been effective in improving the health-related quality of life status of PWE
Kim et al. [57]	1995/1999	Korea	Adult (931/730)	Vehicles for the educational campaign took the form of lectures and small group discussions	Positive trends in public attitudes towards epilepsy were observed after the public education campaign among rural Korean residents

In conclusion, SFA training is crucial as it enhance both basic knowledge and the response to epileptic seizures. Moreover, increased first-aid awareness can reduce unnecessary emergency rooms visits. Targeted training programs significantly benefit both PWE and the broader community. Thus, it is necessary to master the correct SFA measures, and epilepsy is more likely to be treated in primary care settings [58].

## Suggestions

### Training focus of different training objects

To reduce risk of injury and death, the Institute of Medicine (IOM) emphasizes that “anyone can learn basic first aid to help someone when a seizure occurs” [59]. Educational interventions for epilepsy should be tailored specifically for GPs, the general public, and PWE [60]. GPs require training in diagnosis, seizure differentiation, and first-aid techniques. The general public need education on epilepsy, learn to remain calm, and understand safe rescue methods. Targeted campaigns are essential for specific subgroups, such as the elderly and children [61–64], with children aged 7–12 identified as a key demographic for educational programs [50].

### Training methods

For the general public, disseminating efficient, comprehensive, and accurate knowledge of SFA can be enhanced through personalized medical consultations and a collaborative approach to public health education via new media platforms such as TikTok, WeChat, YouTube, and Instagram. These joint interventions not only augment the community's capacity for emergency response but also mitigate the anxiety of bystanders and lessen the psychological strain [65]. The formation of an epilepsy research network, community-based education initiatives, and a paradigm shift towards primary health care for epilepsy have collectively been shown to elevate overall epilepsy awareness and substantially alleviate the seizure burden [48, 66, 67].

The medical staff in some primary hospitals may have limited aid knowledge and skills, but they exhibit a strong willingness to improve. Therefore, training programs for these hospitals should be prioritised to improve overall first-aid capacity [68]. In remote areas, where first-aid training resources may be scarce, online education platforms can provide accessible learning resources and training courses, effectively overcoming geographical barriers [69]. Addressing the scheduling conflicts of busy GPs, providing flexible training schedules, and integrating training content into daily tasks can enhance the appeal and practicality of the training sessions. By engaging in regular training and simulation exercises, GPs can continuously enhance their first-aid skills, enabling them to apply this knowledge more effectively in real-world scenarios [70]. Besides, we recommend that specialists working with PWE provide additional lectures and educational programs. These should include face-to-face sessions to correct misconceptions, such as the incorrect practices of “spill cold water on patient's face to promote wakefulness” and “performing CPR”. Furthermore, we propose that currently practicing GPs receive ongoing training on seizure disorders [71]. This initiative for continued medical education for GPs, along with the integration of detailed modules on basic SFA in the training curriculum for future GPs, should be communicated to those responsible for GPs training and the Ministry of Health.

### Evaluation of training

Through various training methods, we can conduct evaluations from multiple dimensions including knowledge acquisition, skill proficiency, changes in attitude and confidence, and behavioral modifications. In the US, the Epilepsy Foundation has developed seizure training primarily focused on first aid for tonic-clonic seizures. A certification program based on standardized SFA could enhance participants' knowledge and self-efficacy regarding SFA. The outcomes of pilot programs [45] indicate that the program is readily scalable and enjoys high participant satisfaction. Increasing awareness of SFA leads to prompt responses, injury prevention, reduced mortality risk, and ultimately, saves lives. SFA should be recognized as a fundamental component of knowledge for every community GPs.

## The role of the general practitioner

In 2002, the World Health Organization’s Global Campaign Against Epilepsy project (GCAE) in China showed that it was possible to treat epilepsy in primary care settings [48, 72].This requires a collaborative effort between GPs and the public. A partnership between GPs and neurologists, characterized by dual referrals and information-sharing, will ensure that the treatment plan is tailored to meet the specific needs of the patient. GPs shoulder the responsibility of educating patients and their families to help them understand the type of seizure, possible triggers and how to provide first aid and manage them effectively [73]. By emphasizing medication adherence, they can help patients understand the importance of taking prescribed medications to control seizures. Furthermore, GPs can also provide psychological support and raise public awareness of epilepsy through health education initiatives [74]. They can provide training to community members on recognizing the signs of a seizure and taking appropriate first aid measures, including ensuring the safety of the patient, positioning them correctly, and calling for emergency medical services if necessary.

By equipping caregivers and the public with the necessary knowledge and skills, GPs can help reduce the risk of injury and improve the overall management of epilepsy in the community. First-aid training fosters a culture of safety awareness, encouraging both physicians and community members to proactively identify potential hazards and implement preventive measures.

## Development

Inhalable ASMs provide a novel approach for administering emergency treatment during CSs, SE, focal seizures, and acute seizure cluster, with several preparations already available commercially [75–77]. This method is expected to become a key part of emergency care for seizure attacks. Ensuring the rational and safe use of inhaled medications will be a critical focus for future research and practice.

## Conclusions

Community SFA is an important task that impacts the safety and health of patients, as well as the well-being of the community. The whole society should join hands to continuously improve the level and capability of community SFA, minimize the potential risks related to seizures, and thus provide stronger protection and support for PWE.

## Data Availability

Not applicable.

## Abbreviations

ASM: Anti-seizure medication
CAAE: The Association Against Epilepsy of the China
CPR: Cardiopulmonary resuscitation
CSs: Cluster seizures
GCAE: Global Campaign Against Epilepsy
GP: General practitioner
IOM: The Institute of Medicine
PWE: People with epilepsy
SE: Status epilepticus
SFA: Seizure first aid
US: The United States
